# Atypical presentation of bilateral Epstein-Barr virus dacryoadenitis: a case report of corticosteroid resistant orbital inflammation

**DOI:** 10.1186/s12348-023-00349-y

**Published:** 2023-05-12

**Authors:** Charissa H. Tan, Pamela S. Tauchi-Nishi, Adam R. Sweeney

**Affiliations:** 1 University of Hawai’i at Mānoa, John A. Burns School of Medicine, Honolulu, HI USA; 2 Department of Pathology, University of Hawai’i at Mānoa, John A. Burns School of Medicine, Honolulu, HI USA; 3 Section of Ophthalmology, Department of Surgery, University of Hawai’i at Mānoa, John A. Burns School of Medicine, 1380 Lusitana Street, Suite 708, HI 96813 Honolulu, USA

## Abstract

Epstein-Barr virus is a known cause of dacryoadenitis that is typically sensitive to corticosteroid treatment. When affecting the orbit, particularly the lacrimal gland, Epstein-Barr virus may cause chronic proptosis and a bilateral lacrimal mass effect. We provide a case of bilateral Epstein-Barr virus associated dacryoadenitis initially resistant to corticosteroid treatment requiring biopsy and confirmation by polymerase chain reaction of lacrimal tissue. Herein, we discuss the presentation with associated magnetic resonance imaging and histopathologic images, diagnostic dilemma, and treatment of an atypical case.

## Introduction

Dacryoadenitis is characterized by acute or chronic inflammation of the lacrimal gland due to an infectious, inflammatory, or idiopathic etiology. It is an uncommon condition, accounting for 1 in 10,000 new ophthalmic outpatients [1]. Several reports have described dacryoadenitis as one of the only clinical signs of an Epstein-Barr virus (EBV) infection [2, 3]. Patients with systemic EBV infections, such as infectious mononucleosis, have also been noted to have a range of ocular manifestations, including dacryoadenitis [4, 5]. Because of the self-limiting nature of viral dacryoadenitis, patients often experience resolution of their symptoms with symptomatic treatment and do not need to undergo further evaluation. Although EBV has been implicated as the infectious cause in about one-third of recent onset dacryoadenitis cases [1], much of the clinical presentation, radiographic imaging, and histopathology of EBV dacryoadenitis have not been well established. We present an atypical case of chronic dacryoadenitis, initially resistant to standard dose corticosteroid treatments, requiring polymerase chain reaction (PCR) confirmation and extended duration of high dose corticosteroids.

## Case presentation

**Fig. 1:**
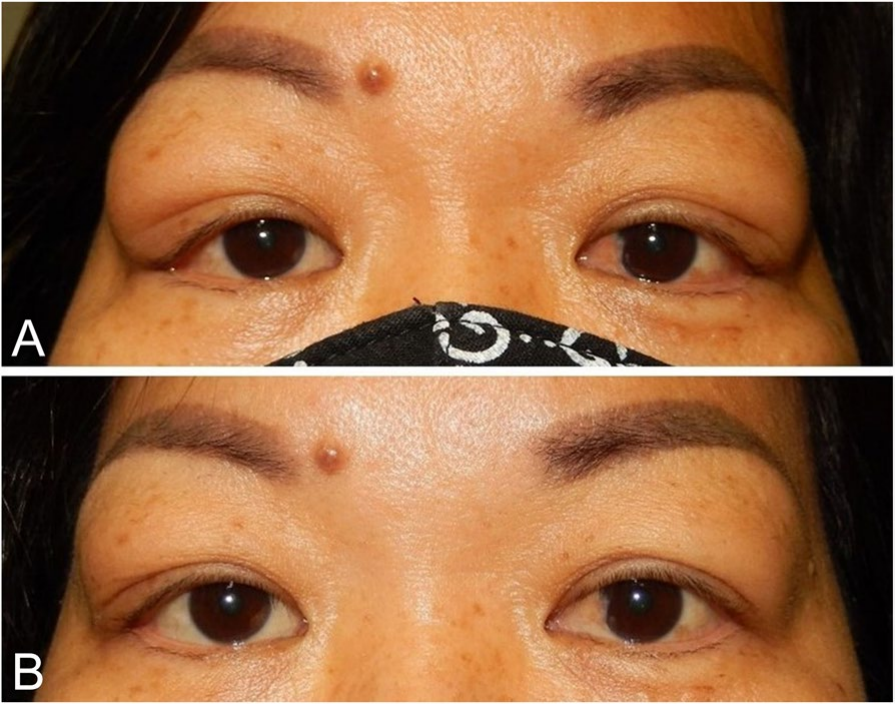
Bilateral EBV-associated chronic dacryoadenitis. Patient (**A**) before and (**B**) after treatment

**Fig. 2:**
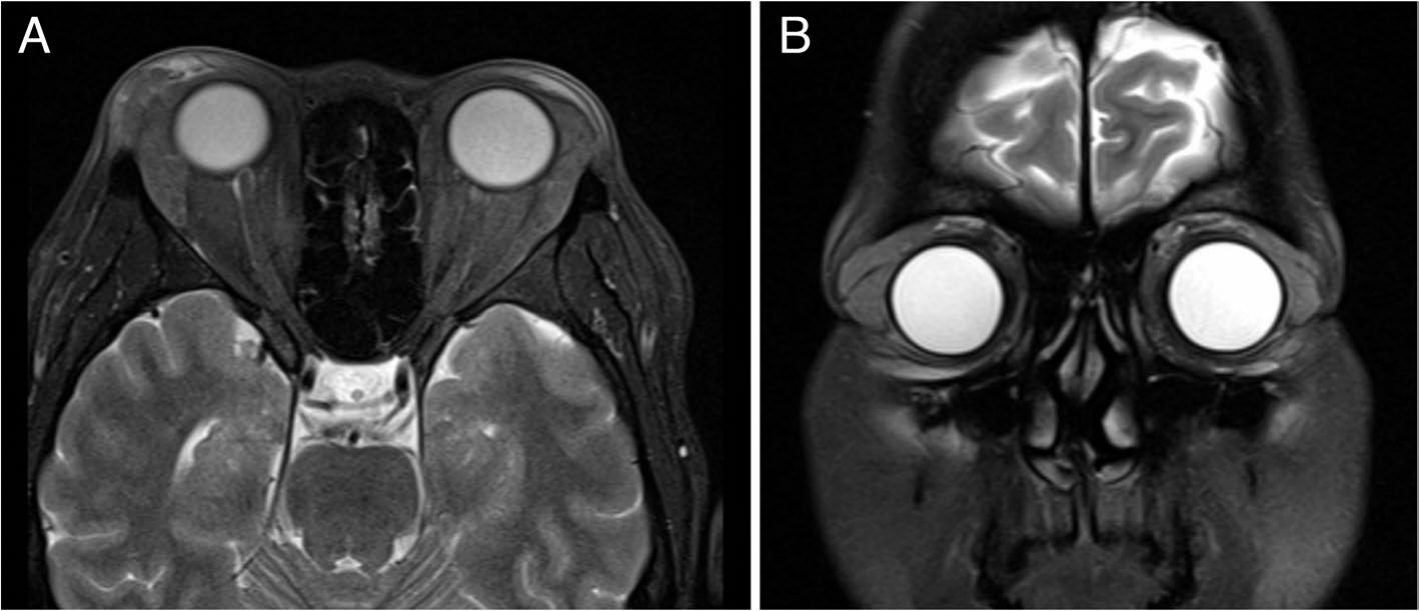
Axial (**A**) and coronal (**B**) T2-weighted MRI demonstrating diffuse enlargement of the lacrimal glands bilaterally with surrounding inflammatory changes. Lesions are softly heterogenous with subtle loculations and do not indent the globe. The lesion reaches far posteriorly in the orbit bilaterally, left greater than right

A 47-year-old female was referred for a 3-month history of worsening bilateral exophthalmos. She had been previously treated by an outside ophthalmologist with an oral prednisone taper starting at 40 mg daily for 2 weeks with no improvement in her disease course and no change in signs or symptoms. When the patient was referred to us, vision was 20/20 in both eyes, pupils were normal bilaterally, and her extraocular motility was full. Hertel’s exophthalmometer measured 19 mm bilaterally. Ocular examination revealed palpable, mobile lesions located in the superotemporal orbits bilaterally (Fig. 1a). On lid eversion, enlarged lacrimal masses were visualized bilaterally. An orbital inflammation laboratory workup including a thyroid panel, amylase, antinuclear antibody, anti-thyroid peroxidase, anti-SSA/Ro, anti-SSB/La, antineutrophil cytoplasmic antibodies, tuberculosis QuantiFERON Gold were obtained to evaluate for autoimmune and infectious etiologies. All returned as normal. Magnetic resonance imaging (MRI) demonstrated asymmetric diffuse enlargement of the bilateral lacrimal glands with a softly heterogenous, loculated appearance and far reaching posterior orbital involvement (Fig. 2).

**Fig. 3:**
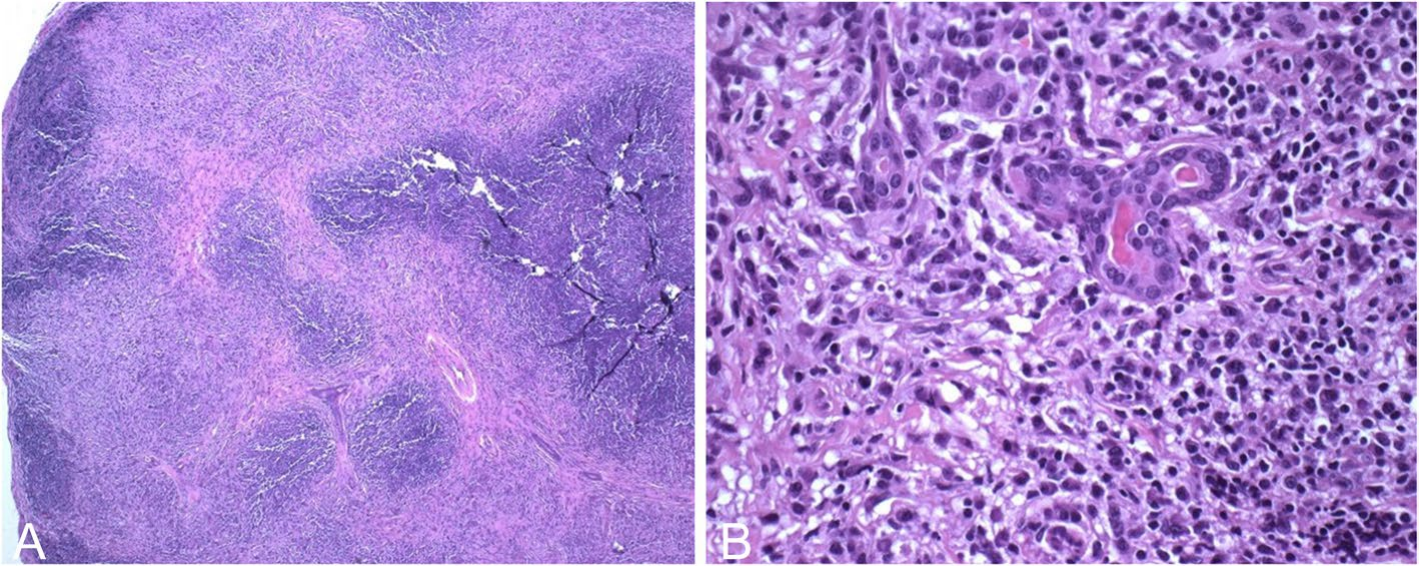
Right orbital mass specimen after staining with hematoxylin and eosin. **A** Low 40X magnification photo displaying chronic inflammation with stromal fibrosis. **B** Higher 400X magnification photo showing sparse remnant lacrimal glands surrounded by a heavy lymphocytic infiltrate

Given the patient had no response to the previously prescribed course of oral glucocorticoid, the decision was made to perform a biopsy of the orbital lesion. The patient underwent a right anterior orbitotomy with biopsy of mass. Standard histochemistry of the specimen demonstrated only chronic inflammation with stromal fibrosis consistent with chronic dacryoadenitis (Fig. 3). Flow cytometry and infectious pathology workup including liquid tumors, adenovirus, syphilis, and tuberculosis studies were unremarkable, ruling out neoplastic, granulomatous, and other infectious causes. Epstein-Barr virus in-situ hybridization was negative, however, EBV deoxyribonucleic acid (DNA) Qualitative Real-Time PCR was detected in the lacrimal tissue.

After diagnosis, the patient was given a single 500 mg solumedrol intravenous infusion followed by an oral prednisone taper starting at 60 mg over one month. The patient rapidly improved while on this extended high dose glucocorticoid regimen. Three months following surgery, the patient’s proptosis had returned to her reported baseline appearance and Hertel’s exophthalmometer measured 15 mm bilaterally (Fig. 1b). All collection and evaluation of protected patient health information was Health Insurance Portability and Accountability Act (HIPAA) compliant, and all research adhered to the tenets of the Declaration of Helsinki.

## Discussion

Signs and symptoms of EBV dacryoadenitis can resolve without treatment within 4 to 6 weeks, but corticosteroids are often used to reduce swelling of the lacrimal gland [1]. In our case, the patient worsened over months even after corticosteroid treatment. Previous reports by Rhem [1], Aburn [2], Marchese-Ragona [3], and Moscovici [4] have not attributed EBV to be resistant to corticosteroids; in fact, patients experienced rapid improvement or resolution within 3 days to 3 weeks of starting treatment. This condition is usually highly sensitive to corticosteroid treatment and the classic response may be considered clinically diagnostic.

Diagnosis of EBV-associated dacryoadenitis has conventionally been made by positive serology [1–3, 6], but there has been an instance where it was made by PCR detection of EBV viral load in blood and tear specimens [7, 8]. Evidence implicating EBV infection as the cause of dacryoadenitis in the aforementioned reports were circumstantial since there was no viral isolation in the lacrimal gland. In another case, lacrimal gland biopsy demonstrated plasma cells that were EBV-positive with in situ hybridization for Epstein-Barr encoding region [9]. Though this substantiates the serologic data, our case highlights the limitations of in-situ hybridization studies alone and suggests PCR testing to be included in the workup. While there have been reports of EBV found in the lacrimal glands of healthy individuals [10], this is the first case report of EBV dacryoadenitis confirmed via PCR of viral DNA in lacrimal tissue.

Biopsy confirmation of EBV dacryoadenitis is best achieved with clear communication with pathology. On biopsy, chronic infectious or inflammatory dacryoadenitis may present nonspecifically with a primarily lymphocytic infiltration or can be mixed with neutrophilic infiltration in a focal, diffuse, or perivascular pattern with possible fibrosis or acinar destruction [11]. As EBV is a ubiquitous virus with approximately 90% prevalence worldwide, definitive diagnostic testing should be done on the lacrimal gland. Coordination of the biopsy specimen for testing for EBV via PCR and in-situ hybridization is needed before tissue processing.

To the authors’ knowledge, while there has been computed tomography (CT) images presented [1, 2, 4, 9, 12]. no prior study has published MRI images demonstrating biopsy-proven EBV dacryoadenitis. On imaging, dacryoadenitis from infectious or inflammatory etiologies commonly present as hyperenhancement of the lacrimal gland affecting the orbital and palpebral lobes without bony changes [12]. Chronic dacryoadenitis is generally seen on imaging bilaterally, while acute or sub-acute infectious dacryoadenitis generally is seen on imaging unilaterally [13]. Combined with the patient’s clinical presentation, the radiographic features of enlarged lacrimal glands bilaterally are classical for the chronic inflammatory changes associated with EBV infection.

In summary, our case adds to the literature further characterizing the radiographic and histologic features of this orbital pathology. Additionally, we provide an example of how in-situ hybridization and PCR testing for EBV may be needed to adequately detect orbital disease. Finally, our case demonstrates a rare case of EBV dacryoadenitis initially resistant to oral corticosteroids requiring a high dose and extended treatment plan.

## Data Availability

Data sharing is not applicable to this article as no datasets were generated or analysed during the current study.

## Abbreviations

EBV: Epstein-Barr virus
PCR: Polymerase chain reaction
MRI: Magnetic resonance imaging
DNA: Deoxyribonucleic acid
HIPAA: Health Insurance Portability and Accountability Act
CT: Computed tomography
Precis: We report a rare case of corticosteroid-resistant bilateral chronic dacryoadenitis secondary to Epstein-Barr virus
